# Prevalence and determinants of pre-adolescent (5–14 years) acute and chronic undernutrition in Lay Armachiho District, Ethiopia

**DOI:** 10.1186/s12939-019-1041-z

**Published:** 2019-09-02

**Authors:** Eleni Belay, Simegnew Handebo, Terefe Derso, Amare Tariku, Mekonnen Sisay

**Affiliations:** 1 Medical Ward, University of Gondar Comprehensive Specialized Hospital, Gondar, Ethiopia; 2 Department of Health Education and Behavioral Sciences, Institute of Public Health, College of Medicine and Health Sciences, University of Gondar, Gondar, Ethiopia; 3 Department of Human Nutrition, Institute of Public Health, College of Medicine and Health Sciences, University of Gondar, Gondar, Ethiopia

**Keywords:** Stunting, Thinness, School aged children, Ethiopia

## Abstract

**Background:**

In Ethiopia it is documented that 16% of all grade repetitions in primary school and 33.9% childhood deaths are associated with undernutrition. School aged children are often omitted from public health research. Thus, the present study was carried out to find out the prevalence and determinants of pre-adolescent (5–14 years) acute and chronic undernutrition in Lay Armachiho District.

**Methods:**

In this community based cross-sectional study, anthropometrics, individual and household characteristics data were collected from December, 2016 to January, 2017. A total of 848 school aged children (5–14 years) were included in the study. Z-scores for height-for-age (HAZ) and body-mass-index-for-age (BAZ) were calculated to illustrate stunting (chronic undernutrition) and thinness (acute undernutrition), respectively with Anthro Plus software version 1.0.4 using the WHO 2007 growth reference standard. Finally, backward stepwise multivariable logistic regression analysis was carried out to identify factors associated with stunting and thinness, individually.

**Results:**

The overall prevalence of stunting and thinness was 35.5 and 9.9%, respectively. The multivariable analysis showed that child age 10–14 years [AOR = 1.58, 95% CI: 1.17, 2.12] and lack of availability of a latrine at home [AOR = 1.60; 95% CI: 1.17, 2.20)] were associated with increased likelihood of stunting. Nevertheless, child’s hand washing practice before eating [AOR = 0.67; 95% CI: 0.49, 0.91] was protective against stunting. Children who consumed diversified foods [AOR = 0.64; 95% CI 0.39, 0.97] were protected from thinness.

**Conclusion:**

In Lay Armachiho district, one-third and one in every ten of school aged children were stunted and thin, respectively. Children age 10–14 years, lack of availability of a latrine at home and hand washing practices before eating were associated with stunting, while only dietary diversity was associated with thinness. Ensuring consistent hand washing practices before eating and ensuring availability of latrine should be improved in the region, which can assist in effectively tackling undernutrition. Finally, dietary diversification should be enhanced to rectify burden of acute undernutrition.

## Background

Child survival, protection and development are the prerequisite for the future development of the nations’ [1]. It is important to ensure optimal child growth and development in order to significantly accelerate economic development of nations [2]. The nutritional status of children is an index of the national investment in the development of future man power; this indicates the changing trend of the nutritional profile of a region [3, 4]. The main nutritional problems facing school aged children are having a low (< − 2 SD) body mass index for their respective age (BAZ)) (acute malnutrition) and low ((< − 2 SD)) height-for-age (HAZ) (stunting) [5, 6].

Stunting in school aged children is associated with poor nutrition in the early childhood years and the degree of stunting tends to increase throughout the school-aged years [7, 8]. However, children can exhibit improvement in their growth and development if their environment improves [9]. Therefore, interventions in school-aged children help to complement efforts in the preschool years to reduce levels of stunting and its related effects on children’s health and education [8].

However, globally malnutrition continues to be a primary cause of poor health and mortality among school aged children particularly in resource limited countries [4, 5]. More than 200 million school aged children are stunted and thin, and unless immediate action is taken, about one billion school aged children will be growing up by 2020 with compromised physical and mental development [9]. In Ethiopia studies conducted in Durbete Town and Addis Ababa city reported that 11.2 and 19.6% of school aged children are stunted [10, 11].

Under-nutrition correlates with decreased ability to fight off infection and poor school performance in children. In addition, undernutrition impaired productivity and earnings during their later life, adulthood period [12–15].

Several studies had identified factors associated with stunting in school-aged children include: male sex, older age group, higher birth order, large family size, low meal frequency, fever, minimal poultry consumption, skipping breakfast, children whose mothers are working, household food insecurity, children who had an unemployed father, and children whose mother had limited education [10, 11, 16–21]. Few studies elicited the correlates of acute undernutrition; having large number of children living the house, unemployed father and inadequate intake of food was associated with being acutely undernourished [16, 17, 21]. However, consumption of animal protein and having a literate household head protected children from acute undernutrition [16].

In Ethiopia, 16 and 34% primary school repetitions and under five mortality respectively were associated with stunting [22, 23]. In spite of this fact, public health interventions do not pay much attention to school aged and adolescents [15].

The majority of the former local studies [24–26] focused on investigating the problem among under five children. There is no previous study conducted regarding the magnitude of acute and chronic undernutrition among preadolescent groups in Lay Armchiho districts. Thus, to fill the knowledge gap, we intended to study stunting, thinness and associated factors among school aged children (5 to 14 years) in Lay Armachiho districts. The findings of this study will help to inform decision for policy and practice.

## Methods

### Study setting and design

A community based cross-sectional study was carried out from December, 2016 to January, 2017 in Lay Armachiho District situated in Amhara region, Ethiopia. Based on the 2007 national census conducted by the Central Statistical Agency of Ethiopia [27], this district has a total population of 157,836 [28]. The district is divided in 26 kebeles *(The smallest administration unit in Ethiopia)*. This region primarily relies on farming to sustain their livelihood.

### Study population and sample size determination

All school-aged (5–14 years) children who lived in the district for at least 6 months were recruited into the study. A single population proportion formula **(n = (Z**_**a/2**_**)**^**2**^ **P (1-P)/d**^**2**^**)** was used to calculate sample size taking into account of the following assumptions; 42.7% as prevalence (P) of stunting in Libo Kemkem District, Ethiopia [16], 95% level of confidence (Z_a/2_)^2^, 5% margin of error (d^2^) and a design effect of 2. Finally, a 15% non-response rate was also added to get the sample size of 869.

A multi-stage sampling was applied to select the samples. Five kebeles were selected out of 26 Kebeles by lottery method. The households with school-aged children in each selected kebele were obtained from the administration offices or from health extension workers of the kebeles. The sample size was distributed to each kebele in proportion to the number of children in each kebele. Systematic sampling technique was carried out after identifying an initial household by lottery method. In addition, if more than one study participant was found in the household, the lottery method was used to select a single study participant.

### Data collection procedures

The structured questionnaire consisted of socio-demographic, health status, dietary practices, and households’ characteristics, and was used to collect the data. The 24 h recall method was used to obtain food consumed in the previous 24 h preceding the survey. Anthropometric data (height and weight) was taken from school-aged children by adhering to the specific standards [29, 30]. Weight was measured to the nearest 0.1 kg with an electronic scale (Seca, 881 U, Germany). Child height was measured to the nearest 0.1 cm using a wooden stadiometer placed on a flat surface. The child stood on the basal part of the device with feet together, without shoes and touched the back of the head, shoulders, buttocks and the heels to the vertical measuring board. Five supervisors and ten data collectors were involved in this study. Intensive training was given to data collectors and supervisors on how to conduct anthropometry measurement and interview techniques by the primary investigator. The questionnaire was pretested. After little modification, it was administered in Amharic, the local language.

### Study variables

Nutritional status of children was assessed using anthropometric indicators, Height-for-Age (HAZ) and Body Mass Index for age (BAZ). Consequently, children having HAZ < − 2SD and BAZ < − 2 SD were considered as stunted and thin, respectively [31]. A single 24-h recall method was used to estimate feeding practice of children and the collected dietary information was transferred to dietary diversity score tool that consisted of seven food groups. Adequate dietary diversity was defined as proportion of children who received food made from four or more food groups in the previous 24-h [32]. Another variable, diarrhea was defined as school children who had three or more loose stools in 24 h in the past 2 weeks from the date of survey [33].

### Data processing and analysis

Data were coded, cleaned, and entered in to Epi-Info version 3.5.3 and then exported to Statistical Package for Social Sciences (SPSS) software version 20 for further analysis. HAZ and BAZ were calculated using Anthro-Plus software version 1.0.4 by the WHO-2007 growth reference standard. Therefore, stunting and thinness were considered as the outcome variables. In the binary logistic regression model, mathematically expressed as; ln (P/1-P) = α + βx P/1-P = e^a + bx^ = Odds d/e = e^a + bx^ and Odds d/e = e^a^ then OR = e^a + bx^/ e^a^ = e^β^ = ln (OR) = β for calculating odds ratio and 95% CI = **e**^**(β** **+** **1.96SEβ)**^ [34]. Backward- stepwise multivariate analysis was used to elicit associated factors of stunting and thinness, separately. Effect size was expressed by odds ratio (OR) with 95% confidence interval. Probability value of type-1 error less than 0.05 was considered statistically significant.

## Results

A total of 848 school aged children (5–14 years) were included in the study with the response rate of 97.6%. Two-third (61.4%) of head of the household were females. Majority household heads were illiterate (42%) and worked as farmers (54.1%) (Table 1). One-thirds of the study participants used protected source of water (67.1%) for household consumption, and latrine was available in 66.6% of the households. The overall prevalence of stunting and thinness was 35.5% [(95% CI: 32.3, 38.7)] and 9.9% [95% CI: 7.9, 11.9], respectively (Table 2).

**Table 1:** Socio demographic characteristics of study participants in Lay Armachiho District, Northwest, Ethiopia, 2017 (*n* = 848)

Variables	Frequency	Percent (%)
Sex of the child		
Male	399	47.1
Female	449	52.9
Age of the child in years		
5–9	510	60.1
10–14	338	39.9
Religion		
Orthodox	829	97.8
Muslim	19	2.2
Residence		
Rural	560	66.0
Urban	288	34.0
Sex of the household heads		
Male	327	38.6
Female	521	61.4
Age of the household heads		
> = 35	258	30.4
36–49	463	54.6
50–65	101	11.9
> 65	26	3.1
Marital status of the head of the households		
Married	673	79.4
Divorced	114	13.4
Widowed	61	7.2
Relationship with the child		
Mother	459	54.1
Father	269	31.7
Grandparent	90	10.6
Others^a^	30	3.5
Occupation of the head of the households		
Merchant	136	16.0
Farmer	459	54.1
Governmental employed	67	7.9
Not employed	186	21.9
Child on education		
No	98	11.6
Yes	750	88.4
Educational level of the head of the households		
No schooling	356	42
Primary	267	31.5
Secondary	225	26.5

^a^Sisters, brothers, aunts and uncle

**Table 2:** Behavioral and households characteristics of study participants in Lay Armachiho District, northwest Ethiopia, 2017

Variables	Frequency	Percent (%)
Time to fetch water		
≤ 30 min	721	85.0
> 30 min	127	15.0
Source of water		
Protected	569	67.1
Unprotected	279	32.9
Water treatment		
No	655	77.2
Yes	193	22.8
Hand washing of the child before feeding		
No	463	54.6
Yes	385	45.4
Availability of latrine		
Yes	565	66.6
No	283	33.4
History of diarrhea in the previous 2 weeks		
Yes	67	7.9
No	781	92.1
Dietary diversity		
Minimum diversity	334	39.5
Poor diversity	514	60.5
Food security status		
Secured	661	77.9
Mildly insecured	108	12.7
Moderately insecured	52	6.1
Severely insecured	27	3.2
Stunting		
Yes	301	35.5
No	547	64.5
Thinness		
Yes	84	9.9
No	764	90.1

Both bivariate and multivariate analyses were carried-out to identify the determinants of stunting and thinness. Primarily, analysis was done to examine the effect of explanatory variables on stunting. Accordingly, the result of bivariate analysis showed that age of the child, availability of latrine, hand washing practice, water treatment, sex and history of child diarrhea had *P*-value < 0.2^1^. Nevertheless, age of the child, availability of latrine and hand washing practice were significantly and independently associated with stunting. The likelihood of stunting was 1.58 times higher among children aged 10–14 years [AOR = 1.58, 95% CI: 1.17, 2.12] compared to their counterparts. The lack of a toilet facility [AOR = 1.60; 95%CI: 1.17, 2.20)] increased the odds of stunting by 1.60 times. Decreased odds of stunting were illustrated among school aged children who had had hand washing habit before eating (Table 3).

**Table 3:** Factors associated with stunting among the study participants in lay Armachiho District, northwest, Ethiopia

Variables	Stunting		COR(95% CI)	AOR(95% CI)
	Yes (#)	No (#)		
Age of the child				
5–9 years	162	348	1	1
10–12 years	139	199	1.50 (1.13, 1.98)	1.58 (1.17,2.12)^*^
Availability of latrine				
No	116	167	1.43 (1.06, 1.92)	1.60 (1.17,2.20)^*^
Yes	185	380	1	1
Hand washing practice of the child before feeding				
Yes	151	308	0.79 (0.59, 1.04)	0.67 (0.49, 0.91)^*^
No	148	237	1	1
Water treatment				
No	242	413	1.33 (0.94, 1.88)	1.24 (0.86,1.81)
Yes	59	134	1	1
Child history of diarrhea in the previous 2 weeks				
No	272	509	1	1
Yes	29	38	1.43 (0.86, 2.37)	0.72 (0.43,1.20)
Sex of the child				
Male	133	266	1	1
Female	168	280	1.20 (0.95, 1.59)	1.21 (0.90, 1.61)

^*^ = Statistically significant at *P* value of < 0.05

On the other hand, the bivariate analysis result of thinness revealed that child age, dietary diversity, age and occupation of the head of households had a *P*-value of < 0.2. However, in the multivariate analysis, only dietary diversity was significantly associated with thinness. Children who consumed diversified diet were found with 34% reduced odds of thinness [AOR = 0.64; 95% CI 0.39:0.97)] (Table 4).

**Table 4:** Factors associated with thinness among school aged children in Lay Armachiho, northwest, Ethiopia

Variables	Thinness		COR[95% CI]	AOR [95% CI]
	Yes(#)	No(#)		
Age of the child				
5–9 years	42	468	1	1
10–14 years	42	296	1.58 (0.96, 2.48)	1.58 (0.98,2.48)
Dietary diversity				
Minimum diversity	26	308	0.66 (0.41, 0.98)	0.64 (0.39, 0.97)^*^
Poor diversity	58	455	1	1
Occupation of the house hold heads				
Merchant	10	126	1	1
Farmer	42	417	1.27 (0.62, 2.60)	1.10 (0.52, 2.31)
Governmental employed	9	58	1.96 (0.75, 5.07)	2.16 (0.81, 5.75)
Not employed	23	163	1.78 (0.82, 3.87)	1.62 (0.73, 3.57)
Age of the household heads				
> = 35	26	232	1	1
36–49	40	423	0.84 (0.50, 1.42)	0.75 (0.44, 1.30)
50–65	13	88	1.32 (0.65, 2.68)	1.08 (0.50, 2.34)
> 65	5	21	2.13 (0.74, 6.11)	2.00 (0.67, 5.96)

^*^ = Statistically significant at *P* value of < 0.05

## Discussion

This study explored stunting, thinness and associated factors among school aged children in Lay Armachiho district. The findings of this current study revealed high prevalence of stunting (35.5%) and thinness (9.9%) among school aged children. The magnitude of stunting in the current study is consistent with a study done in Egypt (34.2%) [18] and Kenya (30.2%) [35]. However, our finding was comparatively higher than a study conducted in Addis Ababa City, Ethiopia (19.6%) [11], a study done in Durbete Town, Ethiopia (11.2%) [10] and the study finding reported from Burkina Faso (8.8%) [36]. The differences might be due to variations in place residence. The participants of the current study were majorly (66%) rural inhabitants compared to the latter three studies. Poor health care access, poor child feeding and caring practice are among a major problem in the rural part of Ethiopia [37].

This implies that unfair access and poor child feeding cause to inequalities in child health outcomes between rural and urban settlements [38].

In the current study, magnitude of thinness (9.9%) was consistent with findings in West Ethiopia (13.2%) [21] and southern Ethiopia (14%) [17], Western region of Nepal (12%) [39] and Burkina.

Faso (13.7%) [36]**.** However, the finding was higher than that of study in Egypt (0.9%) [18]. The discrepancy might be due to the difference in the livelihood of the population; agriculture and industry is the dominant economic sources in Egypt, traditional farming by using an ox is used in the Lay Armachiho district, where the current study took place. Given this information, children of the current study area may not have enough food which increases their risk of acute malnutrition.

In the multivariable analysis the odds of stunting were nearly 1.6 times higher among children aged 10–14 years compared with those aged 5–9 years. Similar local findings were documented in Ethiopia [10, 17]. This may be due to the fact that stunting is a chronic nutritional problem in which, once a child is stunted it difficult to reverse in the late childhood.

In addition, the odds of stunting were 1.6 times higher among school aged children with lack of availability of a latrine at home. Similar results were reported by other local studies [40, 41]. In the absence of a latrine at home open defecation increases contamination of the household’s environment [42]. In support of this evidence study from India confirmed that open defecation increases the likelihood of child stunting [43]. This is presumably related to ingestion of fecal microbes (parasites and bacteria) because of contamination of their finger with fecal matter while playing. This in turn predisposes children with repeated attack of diarrheal diseases which is an immediate cause of malnutrition mainly through malabsorption of nutrients and increased energy and micronutrient requirements [44]. Such explanation suggested the paramount importance of maintaining optimal hygiene and sanitation to curve child stunting. The finding of this study also illustrated the protective effect of hand washing habit of children towards stunting. This report is also supported by the former study from India [45].

Finally, the findings of this study illustrated that dietary diversity, proxy indicator of micronutrient adequacy, of children was significantly associated with thinness. Children who consumed a diverse diet were found to have a lower odds of thinness compared to those who had poorly diversified diet. This report is supported by previous findings elsewhere [17]. Children with a poorly diversified diet and inadequate micro-nutrient content have increased risk of impaired growth and development. This study showed the burden of undernutrition among school children in Lay Armachiho district where evidences are lacking.

## Conclusion

In the Lay Armachiho district, one-third and one in every ten of school aged children were stunted and thin, respectively indicating that the problems are a serious public health concerns. Children age 10–14 years, lack of availability of a latrine at home and hand washing practices before eating were associated with stunting, while only dietary diversity was associated with thinness. Ensuring optimal latrine coverage and hand washing practices are the key to tackle undernutrition. Finally, this study also recommends enhancement of child dietary diversification.

## Limitations of the study

This study tried to address a serious public health issue. However, some limitations, such as measurement errors could be committed while caring out anthropometric measurements. In addition, the study did not test the effect of soil transmitted helminthes on risk of stunting. Due to the cross sectional nature of the study design it could not show the causal relationship between variables. Some of the events involved were happened in the past and hence recall bias can also be another limitation of this study. Furthermore, environmental and genetic factors were not considered in this study which may have an impact on our results.

## Data Availability

Due to ethical restrictions and privacy concerns, a dataset is available upon request from the corresponding author Mekonnen Sisay at mekudesu@gmail.com.

## Abbreviations

AOR: Adjusted odds ratio
BMI: Body mass index
CI: Confidence interval
COR: Crude odds ratio
DDS: Dietary diversity score
SD: Standard deviation
WHO: World Health Organization

## Footnotes

1
*P-value of < 0.2 was chosen as a threshold was by convenience. In addition, majority of researchers used it and scholars recommended it as a cut-off point to control the possible effects of confounders during multivariate analysis.*
